# *QuickStats*: Age-Adjusted Percentages* of Adults Aged ≥18 Years Who Are Current Regular Drinkers of Alcohol,^†^ by Sex, Race, and Hispanic Origin^§^ — National Health Interview Survey, 2016^¶^

**DOI:** 10.15585/mmwr.mm6710a8

**Published:** 2018-03-16

**Figure:**
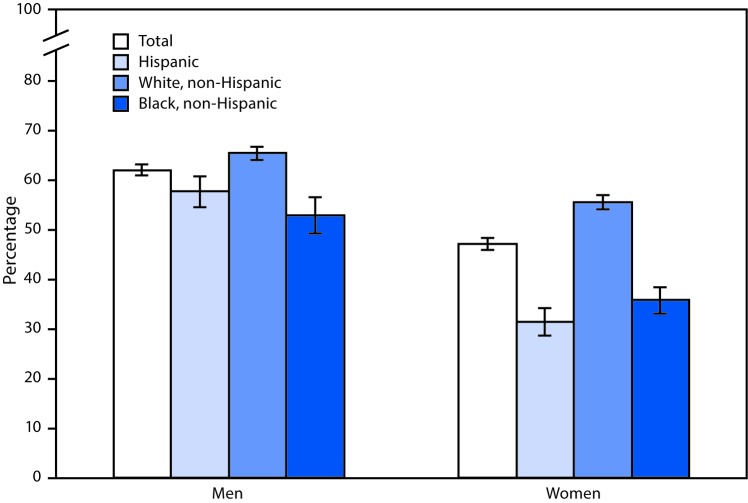
In 2016, men aged ≥18 years were more likely than women to be current regular drinkers of alcohol (62.1% versus 47.2%). Non-Hispanic white men (65.5%) were more likely to be current regular drinkers than Hispanic men (57.8%) and non-Hispanic black men (52.9%). Non-Hispanic white women (55.6%) were more likely to be current regular drinkers than non-Hispanic black women (35.9%) and Hispanic women (31.5%).

**Source:** Tables of summary health statistics for US adults, National Health Interview Survey, 2016. https://www.cdc.gov/nchs/nhis/SHS/tables.htm.

